# Congenital hypoplastic thumbs treated by staged nonvascularized MTP joint transfer for absent MCP joints and abductor digiti minimi tendon transfer for opposition: a case series study

**DOI:** 10.1186/s12891-023-06165-8

**Published:** 2023-03-10

**Authors:** Ramin Zargarbashi, Behnam Panjavi, Mohammadreza Bozorgmanesh

**Affiliations:** 1 Department of Orthopedic Surgery, Tehran University of Medial Sciences, Tehran, Iran; 2 Department of Orthopedic Surgery, Arak University of Medial Sciences, Arak, Iran; 3 Orthopedic Surgeon, Vali-E-Asr Hospital, Tehran University of Medical Sciences, Tehran, Iran

**Keywords:** Hypoplastic thumb, MTP, Metatarsophalangeal joint transfer, Radial club hand, Congenital, Hand, Disorders, Case series

## Abstract

**Background:**

We developed a 2-stage, MTP (metatarsophalangeal) joint- plus ADM (abductor digiti minimi) tendon-transfer, procedure for treatment of hypoplastic thumb. This method is intended to achieve both structural and functional goals of reconstruction. Structurally, it preserves a five-digit hand with minimal donor site complications. Functionally, it provides a functioning opposable thumb.

**Case presentation:**

The case series included 7 patients with type IV hypoplastic thumb. At the first stage non-vascularized joint (not bone) was transplanted. In the second stage abductor digiti minimi tendon was transferred. Patients were followed for a median 5-yr period (range: 37–79 months). Functional outcome was assessed using a modified Percival assessment tool. Participants aged 17 to 36 months at the time of surgery with (2 male, 4 female). All patients were able to grasp large and small objects after the procedure. The thumb tip could actively move to touch the tips of index (2 patients) middle, ring, and little fingers (all patients) in an ulnar ward sequence and vice versa. All patients attained the ability to do lateral, palmar, and tripod pinch. As for donor site complications, none of the patients were found to have difficulty walking or keeping their balance.

**Conclusions:**

An alternative surgical procedure was developed to reconstruct a hypoplastic thumb. We obtained a good functional and cosmetic outcome with few donor site complications. Future studies will be needed to determine the long-term outcomes, to refine the selection criteria and to examine the necessity of additional procedure at the older ages.

**Supplementary Information:**

The online version contains supplementary material available at 10.1186/s12891-023-06165-8.

## Background

Thumb hypoplasia accounts for about 5–15% of congenital hand deformities and is a condition of congenital underdevelopment of thumb [1, 2].

Index finger pollicization has long been the preferred method in thumb reconstruction for the absent thumb or floating thumb [3]. However, the concept of a four-digit hand is prone to stigmatization in many cultures [4]. Drawbacks of pollicization have motivated many surgeons to seek other reconstruction methods with digit preservation. Conventional techniques include second toe–metatarsal bone transfer [5], and distraction lengthening [6]. The toe-to-hand transfer reconstruction techniques share the same limitation of morbidity to the donor site. To tackle this shortcoming Takagi et al. designed an innovative reconstruction method by using nonvascularized metatarsal transfer [7]. Using a similar method, Chow et al. created a functional thumb with grip power and lateral pinch power being 61% and 38% of the normal side, respectively [4]. However, thumb motion is not likely to be restored by this method. Instead of using their thumb, patients will need to coordinate other fingers with a fixed thumb in order to make their hand functional. Directional relationships between the thumb and other fingers will be limited. It is the opposable, and not a fixed, thumb that enables the hand to perform dexterous manipulation of objects. A precisely functioning hand will require well-coordinated digit force vectors [8]. To achieve this, Takagi et al. have developed carpometacarpal arthroplasty of the floating thumb.

Most previous attempts at preserving thumb have been limited to the metacarpal head transfer on the radial side of carpal bones, followed by the muscle transfers. However, arthroplasty in a very young child calls for a complicated procedure and could be burdensome. As such, we have developed an alternative method for the treatment of hypoplastic thumb, where metatarsophalangeal joint rather than metatarsal bone is transferred to the thumb. We hypothesized that joint transfer might improve motion while preserving stability. The most characteristic and elementary movement of the thumb is opposition [9]. As such, tendon transfer has been added to restore opposition of the thumb. Herein, we are reporting the results we obtained from MTP transfer on 7 patients with hypoplastic thumb.

## Methods

### Study design

**Fig. 1:**
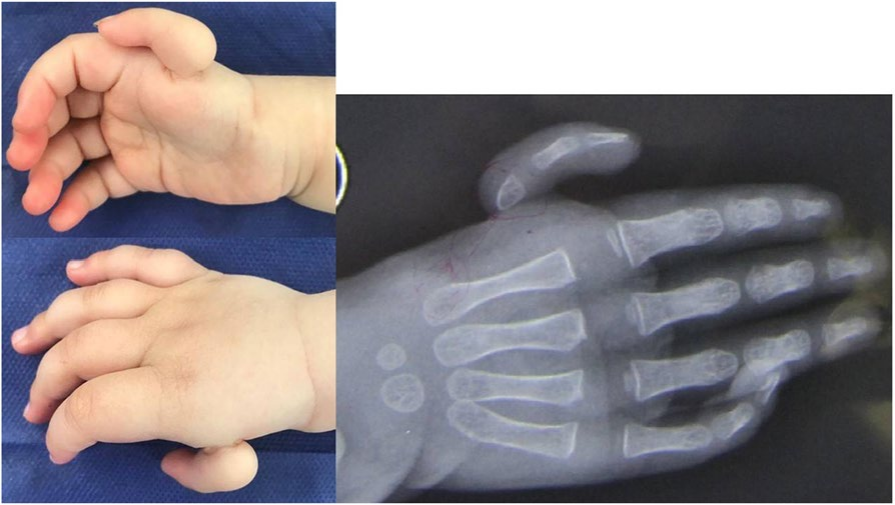
Hypoplastic thumb: an underdeveloped, floating, nonfunctioning thumb with no bony or muscular support, attached to the hand by only skin and soft tissue

This is a report on a small series of cases with hypoplastic thumb (Fig. 1) treated with a metatarsophalangeal transfer. We hereby certify that all applicable institutional and governmental regulations concerning the ethical use of human volunteers were followed during this research. Ethics committee of the Arak University of the medical Science approved the design of the study (IR.ARAKMU.REC.1400.230). Informed written consent was obtained from all participants.

### Study population

We consecutively recruited patients referred to a Children Tertiary Medical Hospital between June 2014 and June 2019.

### Inclusion criteria

Patients were included if they were found to have a modified Blauth’s type 3B and IV hypoplastic thumb as suggested in the literature [10, 11].

### Exclusion criteria

Patients were excluded if their parents did no provide consent.

### Procedures

The procedure included two stages. The first stage could be defined as a non-vascularized joint transplantation. The second stage includes tendon transfer. Below comes the detailed description of the procedure.

**Fig. 2:**
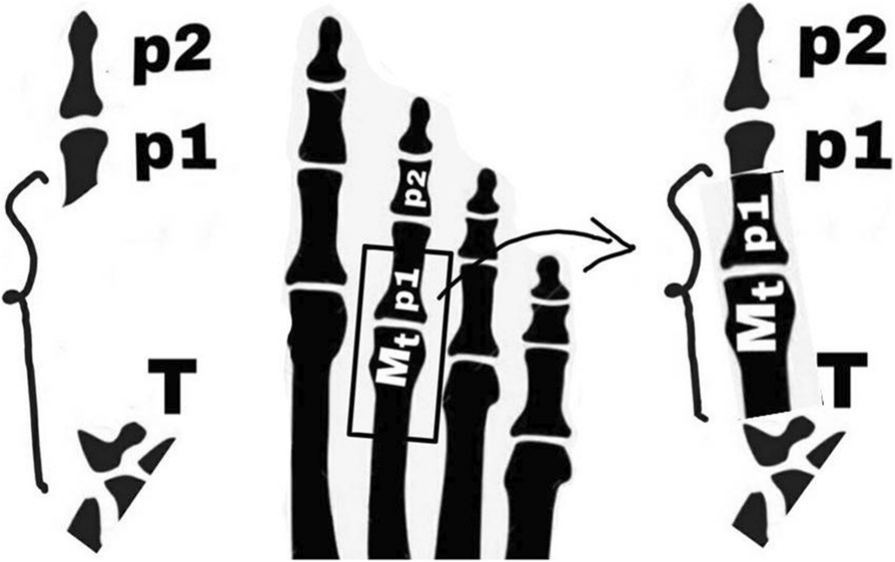
Schematic depiction of the whole procedure

**Fig. 3:**
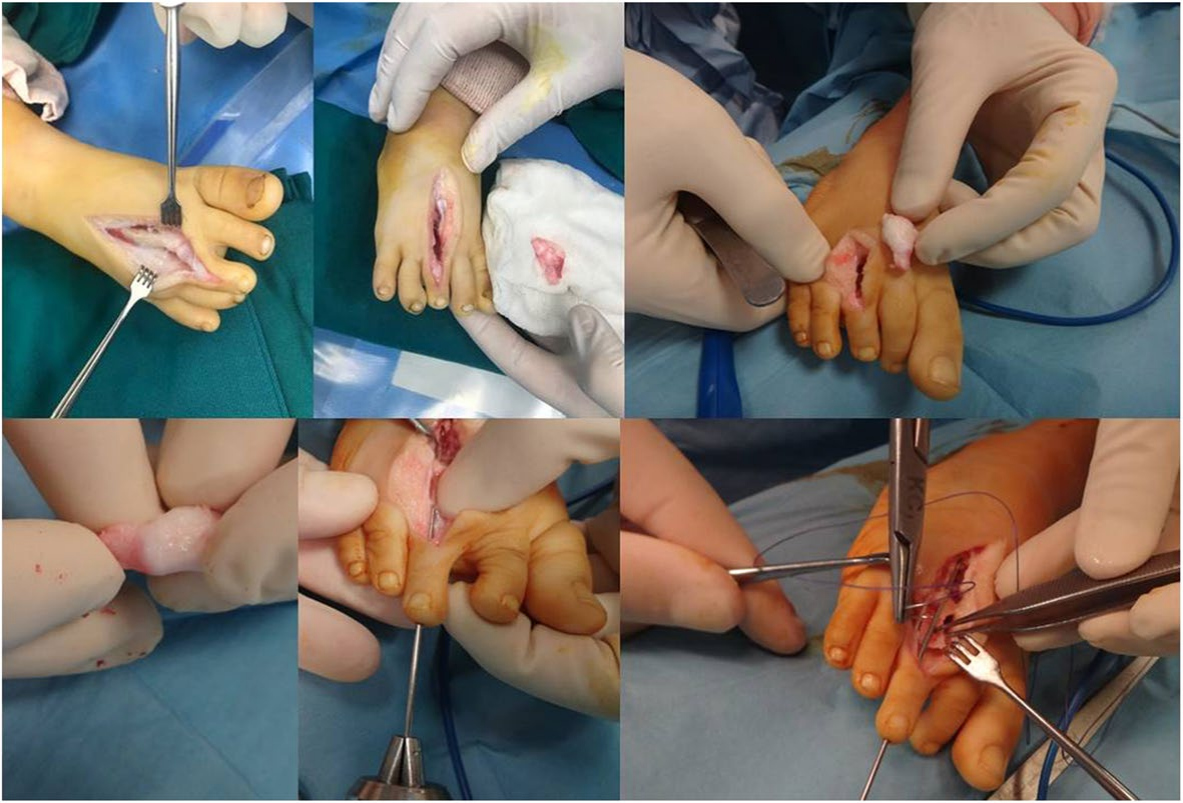
Harvesting metatarsophalangeal joint

**Fig. 4:**
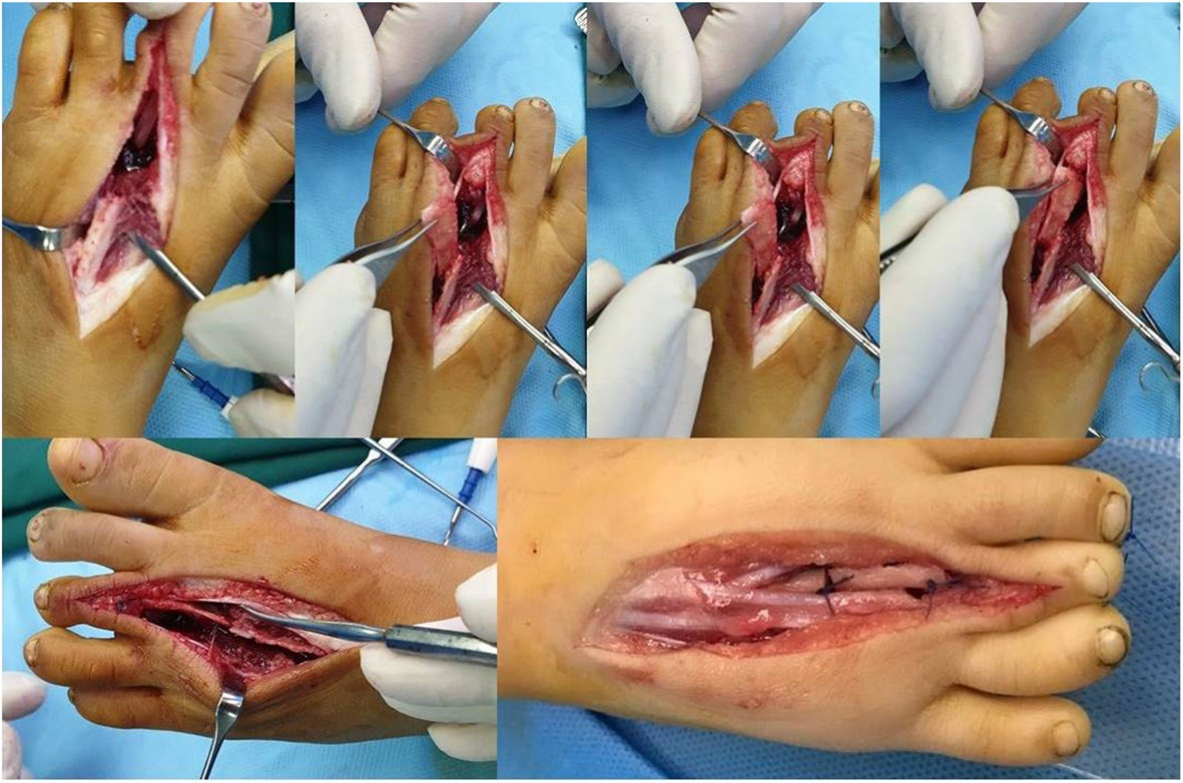
Split-slide metacarpal reconstruction of the foot

The Fig. 2 is a brief, schematic depiction of the whole the MTP transplantation procedure. As shown in Fig. 3, in all patients, we first harvested a non-vascularized joint from the same side 3^rd^ metatarsophalangeal joint. A longitudinal incision was made through the skin and subcutaneous fascia. The extensor tendons were retreated to the sides; neurovascular bundles were explored and protected. The metatarsal bone, metatarsophalangeal joint, and proximal phalanx were explored extraperiosteally. Every precaution was exercised to protect flexor and extensor tendons as well as collateral ligaments. Plantar plate, collateral ligaments and joint capsule were handled delicately while being dissected from neurovascular bundles, flexor tendons and intrinsic muscles. The size of graft (metatarsal and proximal phalanx) was predetermined on the basis of the defect detected in the thumb. Using a small k-wire, we first made holes in the proximal phalanx and on the metatarsal. Then, an osteotomy was performed by connecting holes with an osteotome or surgical blade as appropriate. This way the harvested MCP (metacarpophalangeal) joint included proximal part of the P1 and distal part of the metatarsal bone. We cut flexor digitorum brevis, but preserved extensor and flexor digitorum longus tendon. Plantar plate was meticulously dissected off the plantar fascia. Having preserved the periosteum of the proximal remaining part of the metatarsal, we split it in halves lengthwise. One half was then mobilized, slid distally, and approximated to the remaining part of the proximal phalanx. Vicryl suture was used to fix the remaining part of phalanx distally and remaining half of the metatarsal proximally. Fixation was secured using a longitudinal 1.5 mm k-wire (Fig. 4). In order to avoid shortening, the third phalanx was sutured to the adjacent phalanges using a nonabsorbable suture. This suture is to be left in place and we do not routinely remove it. The wound was closed in multiple layers and a non-weight bearing cast was applied.

**Fig. 5:**
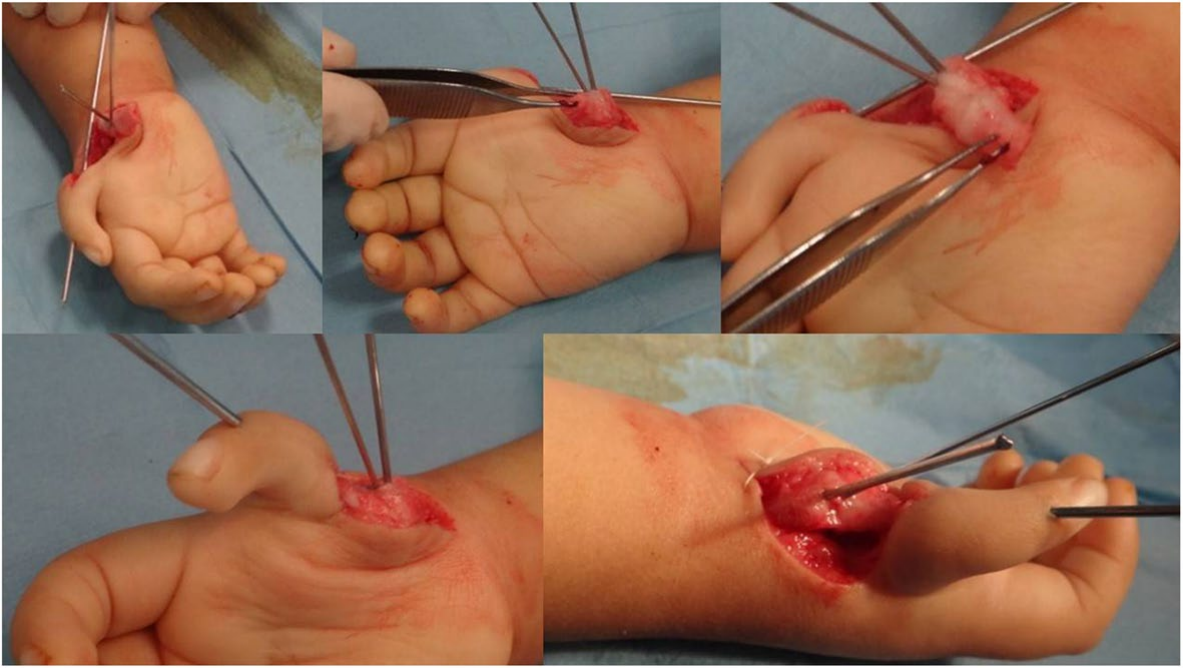
Exploration and preparation of the thumb for metatarsophalangeal joint to be transferred to the hand and transfer and fixation of the metatarsophalangeal graft to the hand. Hypoplastic thumb: an underdeveloped, floating, nonfunctioning thumb with no bony or muscular support, attached to the hand by only skin and soft tissue

Next, a skin incision was made on the dorsolateral aspect of the thumb extending to the base of the first metacarpal. Neurovascular bundles were explored and protected. Adductor pollicis, flexor pollicis brevis, and abductor policies brevis muscles need to be examined at this stage. In our series these muscles were all lacking. Then, we explored the trapezium and the remnant of the first metacarpal. The fibrous tissue between trapezium and fist metacarpal was debrided. The harvested joint was then interposed between the remnant of the first metacarpal distally and trapezium proximally (Fig. 5). None of the patients had a developed MCP joint and the graft was primarily fused to the trapezium to obtain stability at the carpometacarpal junction. In rare occasions, where trapezium is absent, the scaphoid can be used instead (was not the case in our series). The thumb was kept in abduction and extension, a position resembling those of the normal side. Small k-wires were used to fix the joint proximally and distally. Wires were advanced close to, but not across, the joint in order to prevent joint stiffness. The wound was closed in normal fashion. It is of utmost importance to position the metatarsophalangeal joint in accordance with thumb movement such that apposition is possible and hyperextension is avoided. The fixation was secured with a long arm cast. A Z-platy was performed in two cases in order to improve cosmetic results. The K wire tips were left partially exposed outside the skin for easy removal 6 weeks after surgery.

All patients were evaluated within 2 weeks for the first follow-up visit when sutures were removed. A new cast was applied to the upper extremity for 4 weeks.

**Fig. 6:**
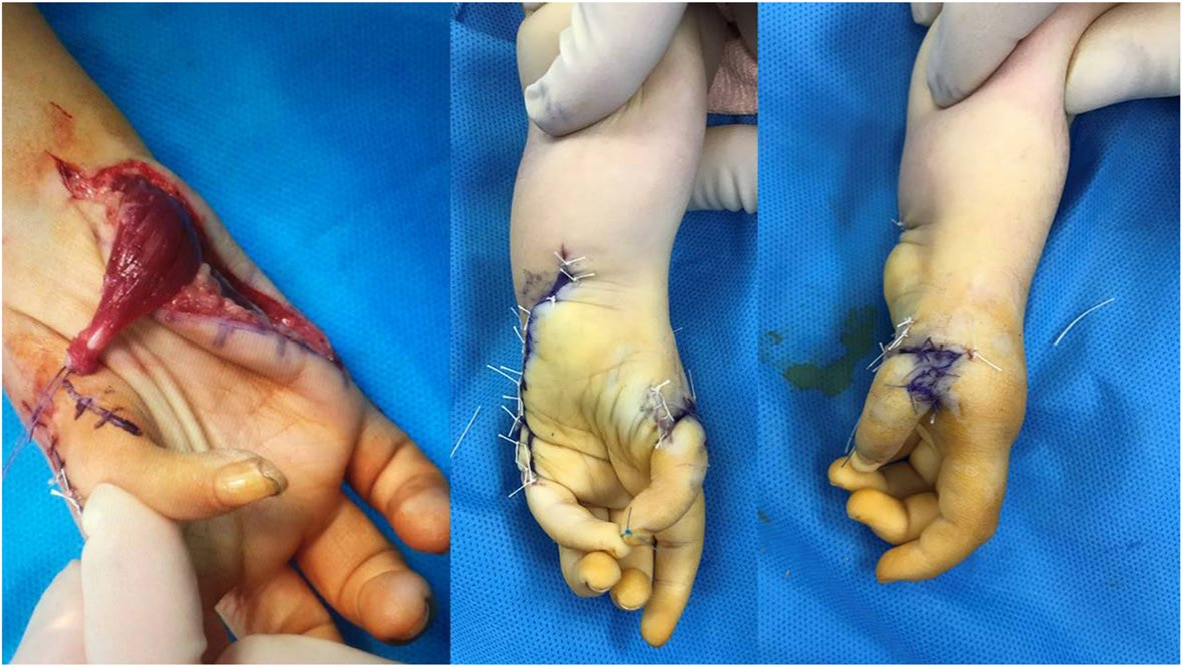
Abductor digiti minimi tendon transfer to achieve apposition

Three months after the MTP transfer abductor digiti minimi (Fig. 6) was transferred to restore opposition as described by Oberlin [12]. Briefly, a longitudinal skin incision was situated on the ulnar border of the hand, starting at the flexion crease of the wrist proximally and extending to the ulnar border of the proximal phalanx distally. A radial incision was made at the base of the thumb, at the level of the metacarpophalangeal joint. The muscle was dissected as long as possible. It was then drawn through a subcutaeous tunnel. The transfer was then fixed on the extensor pollicis brevis or the metacarpophalangeal capsule.

After operation training in the functions under the investigation was begun as early as possible. Daily repetitive and progressive exercises aimed at utilization of gripping ability provided by the operative procedure.

### Outcome

To the best of our knowledge a validated, widely-implemented, objective, tool to evaluate outcome of such procedures in children is yet to be established. However, to enable comparability of our results with those reported previously, we have used a modified version of the assessment tool developed by Percival et al. [13, 14]. We also evaluated whether MTP transfer impacts on hand dominancy and assessed whether patients showed preference for using the hand that was operated on. The sensibility was examined and recorded in terms of having a two-point discriminatory capacity of < 5 mm.

We certify that all applicable institutional and governmental regulations concerning the ethical use of human volunteers were followed during this research. Informed written consent was obtained from all participants parents and the Ethical Committee of Tehran University of Medical Sciences approved this study. Parents of the children of whom pictures or videos are to be published have given their written consent. The study protocol conforms to the ethical guidelines of the 1975 Declaration of Helsinki.

The parents/guardians of the children provided informed written consent to participate. We obtained consent for publication from the parents/guardians of the children included in the study to publish the photos of participants.

## Results

**Fig. 7:**
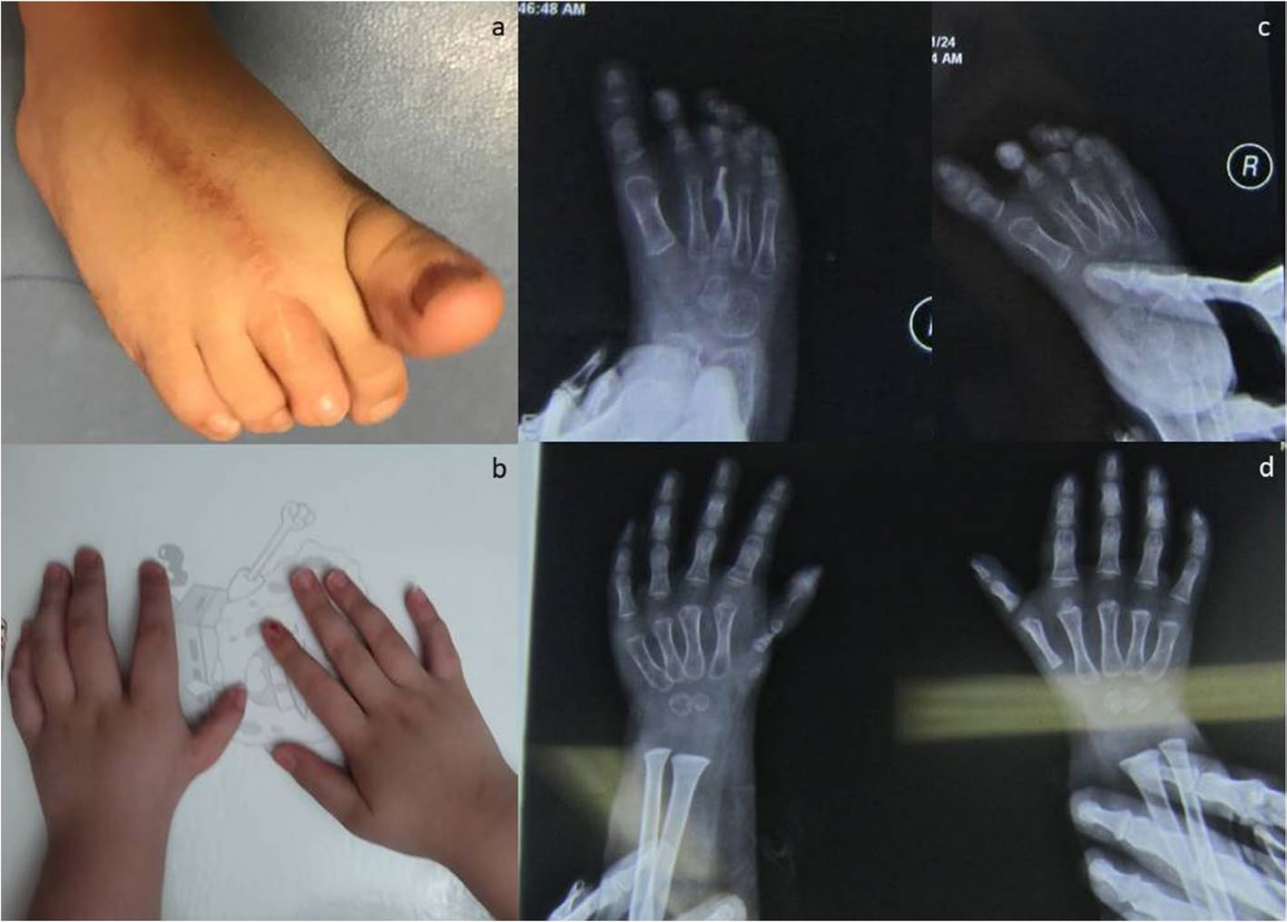
Postoperative appearance of donor site (**a**), reconstructed hand (**b**), and a 5-year postoperative X-ray (**c**)

**Fig. 8:**
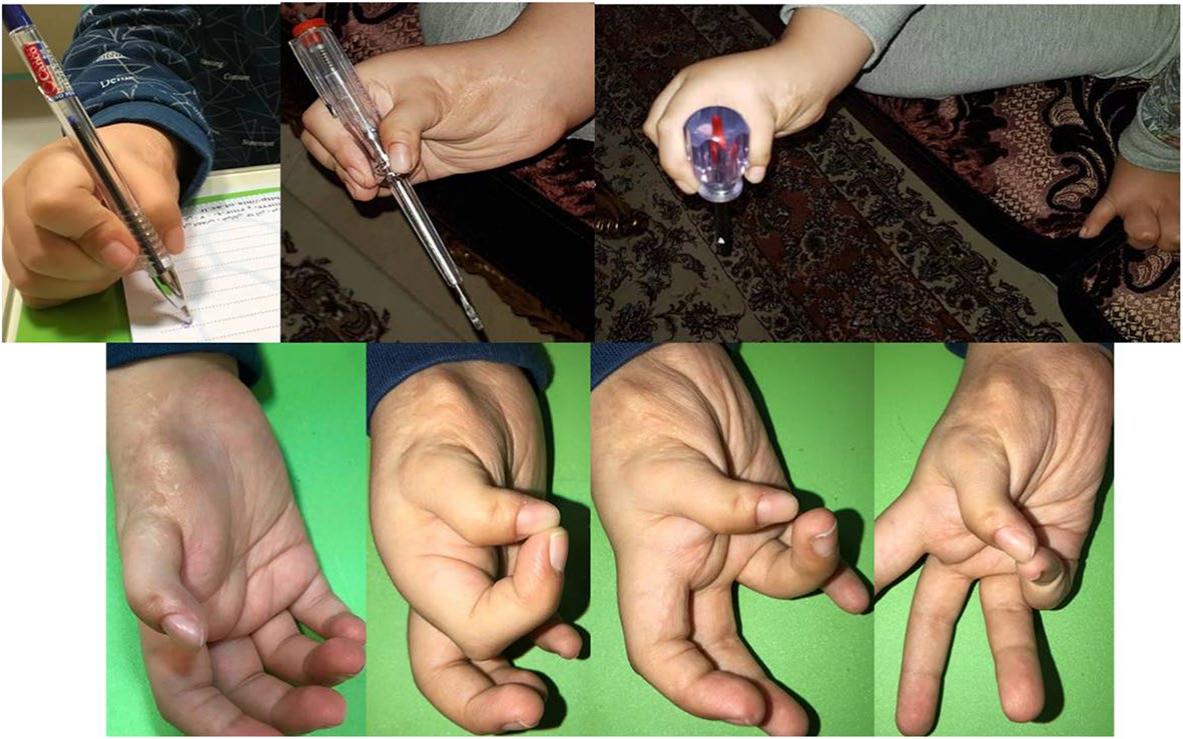
Postoperative restoration of the ability to do different types of grasp and pinch (upper) and postoperative restoration of the ability to do the opposition (lower)

The characteristics of the participants are reported in Table 1. All patients had a type IV hypoplastic thumb. Seven participants (3 male, 4 female) were included in this case series. Mean (SD) age of the participants was 21.0 (7.8) months. The patients were followed for a median period of 57 months (range: 12–79 months). The sensibility was preserved with all patients having a two-point discriminatory capacity of < 5 mm. The donor site and reconstructed thumb are shown at the 5-year post-surgery follow-up (Fig. 7 A&B, respectively). X-rays were taken at both sites to assess for healing (Fig. 7 C&D). At the end of the follow-up, all patients were able to touch the tip of middle, ring, and little finger with the tip of the reconstructed thumb. However, only three out of 7 patients attained the ability to touch the tip of the index finger with their reconstructed thumb (Fig. 8). Similarly, all patients were able to do the key pinch (Video 1), hold small and large objects (Fig. 8). Of 7 patients, 4 were able to draw and write with a pencil (Video 2, 3). True tip pinching was observed in none the patients; however, they were all able to do the palmar pinch and pick up a needle. Supplementary Fig. 1 depicts different types of grasp (large diameter, a; small diameter, b; index extended, c) and pinch (palmar, d; prismatic or tripod or chuck, e; sphere, f). Bilateral hypoplastic thumb was observed in two patients. With regard to change in preferred handedness, only one of the patients was observed to prefer using the hand operated on while before the procedure she preferred using less affected functional hand.

**Table 1:** Characteristics of the participants with type IV hypoplastic thumb treated with MTP joint and ADM tendon transfer

N	Age at surgery	Sex	Deformity in hand	Parent satisfied	Follow-up	Opposition				Grasp		Pinch				2PD^g^ < 5 mm
						I^a^	M^b^	R^c^	L^d^	Large	Small	Tripod^e^	Key^f^	Tip	Palmar	
1	24 mo	M	No	Yes	79 mo	No	Yes	Yes	Yes	Yes	Yes	No	Yes	No	Yes	Yes
2	22 mo	M	No	Yes	67 mo	Yes	Yes	Yes	Yes	Yes	Yes	Yes	Yes	No	Yes	Yes
3	18 mo	F	No	Yes	58 mo	Yes	Yes	Yes	Yes	Yes	Yes	Yes	Yes	No	Yes	Yes
4	17 mo	F	No	Yes	57 mo	No	Yes	Yes	Yes	Yes	Yes	Yes	Yes	No	Yes	Yes
5	14 mo	F	No	Yes	57 mo	No	Yes	Yes	Yes	Yes	Yes	Yes	Yes	No	Yes	Yes
6	36 mo	F	Yes	No	37 mo	No	Yes	Yes	Yes	Yes	Yes	No	Yes	No	Yes	Yes
7	16 mo	M	No	Yes	12 mo	Yes	Yes	Yes	Yes	Yes	Yes	Yes	Yes	No	Yes	Yes
8^ h^	11 mo		-	-	-	-	-	-	-	-	-	-	-	-	-	-

^a^Index

^b^Middle

^c^Ring

^d^Little

^e^Chuck pinch or pencil holding

^f^Lateral pinch

^g^Two-point discrimination

^h^Lost to follow up

### Complications

Parents of patient number 6 were not satisfied with the results. She had a deviation in the phalanx that might need osteotomy. As shown in the supplementary Fig. 2, some degrees of shortening might occur in the metatarsal bone as the child grows older. Having noticed this complication in the first patient, we started to suture the third phalanx to the adjacent phalanges using a nonabsorbable suture. Supplementary Fig. 2 compares cosmetic results for this procedure with and without this step. No patients reported gait or balance problems or difficulty with walking.

Patient number 1, 2, 3, and 8 had PDA, VSD, ASD, and VACTERL syndrome, respectively. The patient number 6 had a phalanx deviation we had to correct with an osteotomy. Patient number 6 encountered venous dysfunction and compartment syndrome after the procedure. The hand compartment fasciotomy incision was done for the patient, but unfortunately bone non-union occurred on the graft site. We grafted bone in the non-union region again. However, the parents of the patient decided to leave the study.

Figure 7 depict the cosmetic result at the implantation as well as the donor site. 

## Discussion

To the best of our knowledge, nonvascularized MTP joint transfer has never been performed for the treatment of hypoplastic thumb. To preserve a 5-digit hand we developed a novel surgical treatment for hypoplastic thumb. Herein, we reported a median 5-year functional and cosmetic outcome of the procedure. The sensibility was preserved in all patients. All patients were able to hold large and small objects at 5-year follow-up and in all patients the ability to touch, with the tip of the thumb, the tip of the middle, ring, and little finger was restored. Key pinch was also restored in all patients.

During the first few years after the surgery, we observed that three of the patients (aged 1, 2 and 3 years) were not able to hold the pencil. We first hypothesized that this might be due to the young age of the patients whose sense of dexterity or ability to follow instructions was as yet underdeveloped. However, when we started to perform the operation on children at younger age, the outcomes appeared to improve. Similarly, we observed that with longer follow-up period of 12 months, they found to be able to hold pencil as they grow older and with more frequent occupational therapy. The importance of these findings is twofold. First it highlights the importance of the follow-up duration for a certain outcome measure. Second, for children with developmental delays due to a physical dysfunction, occupational therapy has been shown to help improve their motor and play skills (3).

The ability to touch the tip of the index finger with the tip of the thumb restored only in three patients. It remains to be elucidated if patients will develop this fine motor function skill as they grow older and with more occupational therapy.

To create a functional thumb for the attainment of a five-digit hand in patients with hypoplastic thumb, Chow et al. suggested a reconstruction method by using nonvascularized hemi-longitudinal metatarsal transfer [4]. Overall, hand function has been reported to be good with no permanent donor site complications [4]. These patients will have to use their fingers very skillfully against a fixed nonfunctioning thumb. In the current study, we have attempted to tackle this limitation by transferring a joint to the thumb and providing a potential for motion [3]. Even these hypoplastic thumbs may well have a normal proximal and distal phalanx structure but may invariably be a bit short and contributing to the overall shortening of the digit.

We performed Z-plasty (first web) when the floating thumb was too small and underdeveloped that there was no sufficient skin available over the thumb to cover the implanted MTP joint. Thumb web is the common site. Widening of these webs by Z plasty will invariably deepen them to some degree. Intentional deepening of the web to make the thumb appear long enough, that is, in taking the thumb web level proximal to mid portion of the axis of the 2^nd^ metacarpal, will require a full thickness skin graft or a full thickness rotation graft with a complex z plasty. Web deepening might be needed later on as the child grows older; however, we did not find it to be necessary at this stage.

For the severely hypoplastic thumb, it might not be feasible to acquire function while preserving the thumb. Takagi et al. have developed carpometacarpal arthroplasty of the floating thumb using metatarsal head replacement. They have reported the functional results to be acceptable with some cases having function limited to simple object holding [7]. If we call our procedure a total joint replacement, Takagi’s is best described as MCP hemiarthroplasty.

### Limitations and strengths

Our findings need to be interpreted in the light of our limitations and strengths. It is plain to deduce that transfer of a non-vascularized small joint would take a long time to vascularize hence a long follow up is vital. There was no control group against which we could compare the outcomes. Older children with short and non-opposing short thumbs may develop thumb web adduction contracture. This needs to be noted, measured, and taken into the equation before ADM transfer. Hypoplastic thumbs we operated on were, generally, underdeveloped, floating, nonfunctioning thumbs with no bony or muscular support. They were attached to the hand by only skin and soft tissue. After MTP transfer and before ADM transfer, however, contracture could be an issue. Mindful of these considerations, a surgeon needs to be prepared to supplement the basic procedure with additional stages pro re nata. Additional stages include rotational flap for the first web space widening and deepening, further tendon transfer to strengthen adduction of thumb and/or abduction of index finger (for Key pinch) and flection of IP joint (for tip pinch). Web deepening might, still, be needed later on as the child grows older; however, we did not find it to be necessary at this stage. For Manske type 4 hypoplastic thumbs, using local flap or free vascularized composite flap might be necessary. Not only does it enable us to close the wound without tension, but also improves the appearance of the thenar eminence area [15]. It is arguable that the trapezium/trapezoid might have been hypo-plastic could be existed which is irrelevant to the severity of the hypoplastic thumb [16]. An anonymous referee has suggested that for this kind of cases, harvesting the metatarsal only is preferred to harvesting the whole MTP joint. There is at least a theoretical concern that MTP joint might lose its motion over time. A longitudinal study could assess for this possibility. The procedure was more likely to be successful if performed at very young age. If the procedure is to be performed at older ages intraoperative in vivo measurement of the defect could be more accurate than radiological preoperative planning. Considering the very young age of the patients [mean (SD), 22 (8)] we were not able to record any quantitative measure of pinch.

The strength of the innovated procedure proposed herein lies in the fact that transferring joint, at least theoretically, makes the restoration of fine motor motion more likely as compared to the procedures that include transferring only metatarsal bone without any joint. Furthermore, sensory function is more or less preserved in these procedures as compared to vascularized toe-transfer. As such, this procedure may be more likely to promote brain plasticity [17]. Furthermore, the procedure is more cosmetically acceptable than the pollicization because it does not negate the concept of a five-digit hand (Fig. 7). There is an extensive literature on non-vascularized toe phalanx transfer to the hand. Most authors have concluded that these do not grow much. As the rest of the hand grows, the small fingers becomes less effective [18, 19]. Our procedure might offer potentials for future growth since the growth (epiphyseal) plate of the implanted metatarsal bone is preserved. Whether such a theoretical potentiality could be tapped into practically remains to be determined with longer follow-up. Recently, Liu et al. have used non-vascularized transfers [20]. They have cautioned about the growth potential of the transplanted bone. However, in a cohort of younger patients (aged 6 to 18 months of age), Goldberg and Watson observed that in grafts harvested with an intact periosteum, 91% of the epiphysis remained open, and growth averaged 83% to 100% of the contralateral toe [21]. Similarly, Buck-Gramcko [22] reported that the epiphysis remained open in 66% for non-vascularized grafts for children between 7 and 18 months of age. As such, we could suggest that if non-vascularized transfers are performed in children under 2 year an acceptable amount of growth could be expected [20]. The donor site morbidity may become more prominent in the long term. As such, longer follow-up will be required to determine if the current procedures will be able to prevent donor site morbidity.

## Conclusion

An innovative surgical procedure was developed to reconstruct hypoplastic thumbs. This method is intended to achieve both structural and functional goals of reconstruction. All patients were able to touch the tip of middle, ring, and little finger with the tip of the reconstructed thumb. Most of the patients were able to draw and write with a pencil. True tip pinching was observed in none the patients; however, they were all able to do the palmar pinch and pick up a needle. Future studies will be needed to determine the long-term outcomes, to refine the selection criteria and to examine the necessity of additional procedure at the older ages.

## Supplementary Information

**Additional file 1: Supplementary Figure 1.** Different types of grasp (large diameter, a; small diameter, b; index extended, c) and pinch (palmar, d; prismatic or tripod or chuck, e; sphere, f).

**Additional file 2: Supplementary Figure 2.** Comparison of cosmetic results between procedures with (a) and without (b) suturing the third phalanx to the adjacent phalanges. As shown in the figure (b), the shortening occurs in the third metatarsal bone as the child grows older. Having noticed this complication in the first patient, we started to suture the third phalanx to the adjacent phalanges using a nonabsorbable suture. As shown in the figure, some degrees of shortening might occur in the metatarsal bone as the child grows older. Having noticed this complication in the first patient, we started to suture the third phalanx to the adjacent phalanges using a nonabsorbable suture.

**Additional file 3.**

**Additional file 4.**

**Additional file 5.**

## Abbreviations

MTP: Metatarsophalangeal
ADM: Abductor digiti minimi
MCP: Metacarpophalangeal
IP: Interphalangeal
CMC: Carpometacarpal
TMC: Trapeziometacarpal
