# Supplementary figures and images for: Congenital hypoplastic thumbs treated by staged nonvascularized MTP joint transfer for absent MCP joints and abductor digiti minimi tendon transfer for opposition: a case series study
Source: BMC Musculoskelet Disord. 2023 Mar 10;24:179. doi: 10.1186/s12891-023-06165-8 (PMC9999557; doi:10.1186/s12891-023-06165-8)

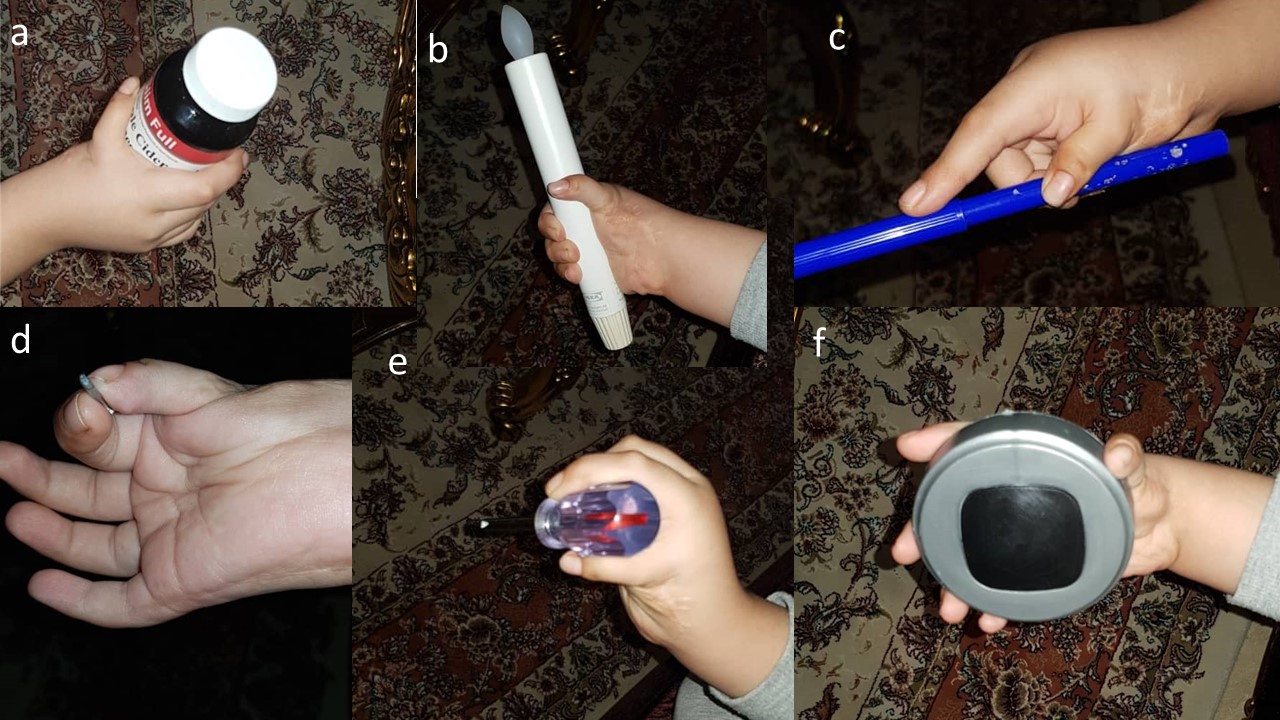

Supplement: Supplementary file 1 — Additional file 1: Supplementary Figure 1. Different types of grasp (large diameter, a; small diameter, b; index extended, c) and pinch (palmar, d; prismatic or tripod or chuck, e; sphere, f). [file 12891_2023_6165_MOESM1_ESM.jpg]

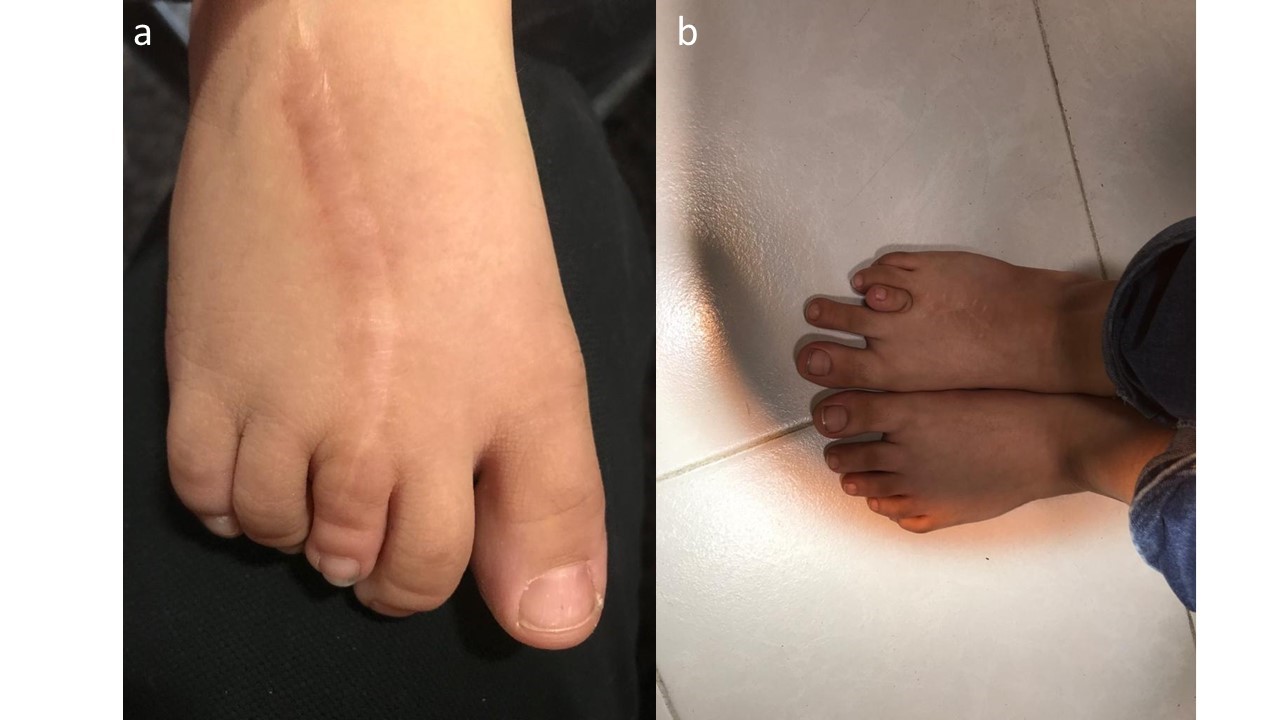

Supplement: Supplementary file 2 — Additional file 2: Supplementary Figure 2. Comparison of cosmetic results between procedures with (a) and without (b) suturing the third phalanx to the adjacent phalanges. As shown in the figure (b), the shortening occurs in the third metatarsal bone as the child grows older. Having noticed this complication in the first patient, we started to suture the third phalanx to the adjacent phalanges using a nonabsorbable suture. As shown in the figure, some degrees of shortening might occur in the metatarsal bone as the child grows older. Having noticed this complication in the first patient, we started to suture the third phalanx to the adjacent phalanges using a nonabsorbable suture. [file 12891_2023_6165_MOESM2_ESM.jpg]
